# Librarian-Led Assessment of Medical Students’ Evidence-Based Medicine Competency: Facilitators and Barriers

**DOI:** 10.5334/pme.1145

**Published:** 2024-03-05

**Authors:** Joey Nicholson, Caitlin Plovnick, Cees van der Vleuten, Anique B. H. de Bruin, Adina Kalet

**Affiliations:** 1 NYU Health Sciences Library, NYU Grossman School of Medicine, NYU Langone Health, US; 2 NYU Health Sciences Library, NYU Grossman School of Medicine, NYU Langone Health, New York, US; 3 Department of Educational Development and Research, Faculty of Health, Medicine and Life Sciences, Maastricht University, Maastricht, NL; 4 School of Health Professions Education, Faculty of Health, Medicine and Life Sciences, Maastricht University, Maastricht, NL; 5 Robert D. and Patricia E. Kern Institute for the Transformation of Medical Education, Medical College of Wisconsin, Wauwatosa, Wisconsin, US

## Abstract

**Introduction:**

We must ensure, through rigorous assessment that physicians have the evidence-based medicine (EBM) skills to identify and apply the best available information to their clinical work. However, there is limited guidance on how to assess EBM competency. With a better understanding of their current role in EBM education, Health Sciences Librarians (HSLs), as experts, should be able to contribute to the assessment of medical student EBM competence. The purpose of this study is to explore the HSLs perspective on EBM assessment practices, both current state and potential future activities.

**Methods:**

We conducted focus groups with librarians from across the United States to explore their perceptions of assessing EBM competence in medical students. Participants had been trained to be raters of EBM competence as part of a novel Objective Structured Clinical Examination (OSCE). This OSCE was just the starting point and the discussion covered topics of current EBM assessment and possibility for expanded responsibilities at their own institutions. We used a reflexive thematic analysis approach to construct themes from our conversations.

**Results:**

We constructed eight themes in four broad categories that influence the success of librarians being able to engage in effective assessment of EBM: administrative, curricular, medical student, and librarian.

**Conclusion:**

Our results inform medical school leadership by pointing out the modifiable factors that enable librarians to be more engaged in conducting effective assessment. They highlight the need for novel tools, like EBM OSCEs, that can address multiple barriers and create opportunities for deeper integration of librarians into assessment processes.

## Introduction

Assessment of a physician’s evidence-based medicine (EBM) ability has traditionally been tests of knowledge and skill [1,2]. Meanwhile in medical education, student assessment is evolving from focusing on knowledge to embracing the complexity of competence or the ability to apply knowledge to improve actual practice. While there are existing competency frameworks for EBM [3], there is limited guidance on how competency in this area should be assessed or who should be the assessors [2,4]. Health sciences librarians (HSLs), as primary instructors of this domain, should be, but rarely are fully integrated into the program of assessment for medical students [5,6,7]. Recent qualitative studies on HSLs emphasize the challenges and opportunities faced both in their job tasks and educational roles [6,8]. However, HSL involvement in assessment continues to be under-developed and under-utilized. Understanding how librarians can contribute to the EBM competency assessment of future physicians will advance the modernization of that assessment and thereby improve future patient care.

EBM as a construct has several overlapping definitions with different steps and varied implications for clinical practice. For the purposes of this research, we used the definition of EBM from the 2005 Sicily Statement. This statement reflects the original steps of EBM from the early 1990s [9], and further elaborates providing explanation and supporting evidence behind the importance of each step [10]. They define the five steps of EBM as Ask, Acquire, Appraise, Apply, Assess. Within this definition and for the purposes of this research, we focused on Ask, Acquire, and Appraise - the EBM steps that HSLs most commonly teach [5]. Despite these being the most taught steps by librarians, there is a need for rigorous librarian-led assessment, including demonstration and observation of behaviors and incorporation into existing assessment process for medical students [5].

While many HSLs assess medical student EBM skills, these assessments tend to be based solely on multiple choice questions or essay-style written exams and run parallel to and therefore are poorly integrated with other assessments [5]. As a result, current EBM assessments do not provide students formative feedback on actual performed behaviors embedded within authentic clinical activities. This means students’ learning is suboptimally reinforced and clinical competence is not built [1,2,11,12]. HSLs need new tools to assess student EBM behaviors [1]. These new tools must be pragmatic, and able to fit into the contexts and constraints in which librarians typically work in medical schools.

Going beyond multiple choice, short answer, and essay, the use of an Objective Structured Clinical Examination (OSCE) format to assess various steps of EBM has been tried. However, from the HSL perspective it has not always been successful. Most frequently, EBM OSCEs focus primarily only two EBM steps: Appraise and Apply [13,14,15]. A recent OSCE developed by Kumaravel et al, includes the first two steps: Ask and Acquire [13] in their EBM OSCE station which uses a talk-aloud procedure and requires learners to use PubMed. Despite yielding a reliable assessment score, the development of this station was not guided by information-seeking behavior frameworks nor with the input of librarians providing formative feedback, rendering it overly prescriptive and unaligned with real-world practices. To advance HSL ability to assess medical students in a rigorous way, another tool and process to assess EBM behaviors via direct observation by a HSL within an OSCE was developed [16,17]. This process involves an HSL remotely observing and rating the medical student’s patient-specific clinical question formulation and literature-based evidence search strategy. Rating is completed using a behaviorally-anchored and theory-based rubric [17].

Existence of this novel OSCE and rubric, however, does not answer questions about implementation, such as capacity or institutional support for HSLs to be involved more deeply in assessment. With new tools available to use in EBM assessment, it is now necessary to better understand the contexts in which HSLs conduct assessments of medical students. Through a more nuanced understanding, we can better inform dissemination and uptake of assessment practices more deeply embedded and aligned with existing medical student assessments.

The purpose of this study is to explore the HSLs perspective on EBM assessment practices, both current existing practices and possible future directions. Through our analysis, we aim to identify common barriers to successful EBM assessment and provide potential solutions that can be employed across institutions.

## Method

### Data collection & analysis

In April 2022, we emailed 15 HSLs to participate in focus groups about their experiences in assessing EBM at their institutions. We offered each participant a $25.00 incentive. In addition to their expertise in teaching and assessing EBM, participants all shared a common experience rating medical student performance via video observation in the OSCE described above.

These 15 HSLs had all been engaged as a convenience sample in Spring 2020 to participate as student raters at Medical College of Wisconsin. This OSCE station was part of an immersive multi-station readiness-for-residency OSCE, Night-onCall [16]. The librarians were all trained to use the previously described rubric to reliably assess student performances [17]. Training included a 45-minute orientation to the background of the rubric conducted by JN and required HSLs to engage with a set of 10 practice videos to practice rating and calibrate their ratings. While some participants had implemented this EBM OSCE at their institution, this was a new assessment method for most. Despite being a convenience sample, recruitment of HSLs was targeted to ensure a range of years of experience, region of the US, type and size of medical school, and current involvement in EBM assessment.

The focus group guide was developed based on a literature review and author expertise and refined through two rounds of testing and feedback from all co-authors. The guide invited participants to describe the current state of their roles in teaching and assessing EBM at their home institutions, gaps and opportunities in EBM assessment. To elicit a deeper understanding of possible implementation of future EBM OSCEs, we included prompts on the usefulness and feasibility of this OSCE format as well as barriers perceived to instituting similar initiatives at their own institutions. See Appendix 1 for the focus group guide. We (JN and CP) facilitated 1-hour focus groups. All focus groups were audio recorded and transcribed verbatim for analysis.

Analysis of the focus group transcripts followed the stages in reflexive thematic analysis as defined and revised by Braun and Clarke [18,19,20]. We used reflexive thematic analysis because of its flexible and interpretive approach to understanding and describing the meanings and patterns held in individual viewpoints about themselves, their work, and the contexts and structures in which they work. Initially, two authors (JN and CP) familiarized themselves independently with the content by reading through and doing an initial round of light coding of the transcripts. Following familiarization, they moved into coding by first meeting to compare and discuss their initial insights. Then, they progressed to independent deductive coding using an agreed upon set of 39 base codes developed following their familiarization. See Appendix 2 for the codebook. In addition to this base set of codes, the authors moved forward with an openness to adding, changing, or discarding codes as they continued to work with the material.

Following the familiarization, generation of codes, and deductive coding processes, JN and CP constructed themes. To construct themes, JN and CP used the codes as clusters of meaning and extracted data on overall frequencies and code overlap. This data was used as a basis for JN and CP to independently think about construction of themes and what meaning they each found in the data so far. Finally, JN and CP revising and defining themes over several rounds of discussion. These themes were reviewed by all authors, who were able to bring their expertise in medical education, assessment, and practicing EBM as a clinician into the conversation. These discussions informed further refinement and comparing back to codes to ensure adequate coverage. Once themes were finalized, they were divided into four categories to highlight the relevant functional area where the theme applies. The categories also serve to enhance clarity since themes like limited time are highlighted both as a librarian factor (individual time to do the necessary work) and as a curricular factor (time allotted within a curriculum). The Dedoose Desktop App, version 9.0.54 (SocioCultural Research Consultants, LLC, Los Angeles, CA, USA) was used to store and code the transcripts.

### Reflexivity

We acknowledge that the conduct and results of this study are co-constructed among the authors and participants. The main focus group facilitator (JN) has a master’s degree in library and information science and public health. He has been a HSL teaching and assessing of EBM for 18 years. This background allowed him to participate in the focus groups as both facilitator and peer, and participants were comfortable with candidly sharing their experiences. The focus group co-facilitator (CP) has a master’s degree in library and information science and has been working as an instruction librarian for 12 years. While relatively new to EBM assessment, she has been active in information literacy and evidence-based practice assessment for Nursing and Allied Health programs throughout her career. Her background informed her understanding of the focus group discussion and helped set the participants at ease knowing they could discuss any topic using jargon without being judged or having to explain.

## Results

Twelve librarians from 11 different medical schools participated in one of three focus groups (4–5 participants each). See Table 1 for a description of the HSLs and their institutional contexts. Participants represented a range of experience in assessing EBM with majority of their assessment experience occurring in a classroom setting in the first 1–2 years of medical school. All participants had experience providing EBM feedback to graduating medical students using the EBM OSCE, but only three (25%) represented institutions that formally administer this assessment activity for students. In describing current and aspirational EBM assessment activities, themes fell into four categories: librarian factors, administrative factors, curricular factors, and medical student factors.

**Table 1:** Characteristics of health science librarian (HSL) participants and their institutional contexts.


PARTICIPANT CHARACTERISTICS	N(%)

Years of Experience	

<5	1 (8.3)

6–10	3 (25)

11–15	3 (25)

16–20	3 (25)

>21	2 (16.7)

Gender Identity	

Female	11 (91.7)

Male	1 (8.3)

Geographic Region	

Northeast	5 (41.7)

South	3 (25)

Midwest	3 (25)

West	1 (8.3)

Type of Medical School	

Public	6 (50)

Private	6 (50)

Size of Medical School Class	

<100	1 (8.3)

101–150	4 (33.3)

151–200	2 (16.7)

>201	5 (41.7)

Feedback Given on EBM	

Pre-Clerkship	8 (66.7)

Transition to Clerkships	2 (16.7)

Clerkships	4 (33.3)

None	2 (16.7)

Institutional Participants in Night onCall	

Yes	3 (25)

No	9 (75)


### Librarian Factors

#### Professional Role

All librarians reported that assessment of EBM is a part of their professional identity and an integral component of their role in educating medical students. For example, one participant noted: ‘If we’re teaching it, we should be assessing it’ (L4). Librarians report dedicating a lot of time and effort in conducting assessments of some kind, usually essay style assignments with written formative feedback. However, this style of assignment is becoming increasingly unpopular: ‘Written feedback for written assignments, although this might change because the students are not liking it very much’ (L3). Despite feeling strongly that assessment was a task they should be involved in, factors outside of their control, like limited time, often interfered in their ability to do this effectively. The long-form written assessment is not only time intensive to assess, but librarians also expressed a desire to use updated, more relevant assessment tools:

‘the longer, the validated instruments, so Berlin and Fresno and whatever else is out there - there have been some validated search strategy evaluation tools over the years, but they’re all kind of old and they were designed at a time when you had to do everything with PubMed, like UpToDate really was kind of new, DynaMed certainly didn’t exist, and they - it just doesn’t reflect, as you said, what they’re doing at the bedside these days’ (L4).

#### Novel EBM Assessment Tool

All librarians expressed appreciation for the novel format of the OSCE and the opportunity to assess observed student behaviors instead of written descriptions. One participant noted, this can also help connect the students to learning the content in a more meaningful way:

‘I think there’s a major advantage to it because when you’re dealing with, particularly first and second years, they don’t have anything to hang it on, they don’t understand this is all connected: this will be patient care, this helps make your decisions later, this helps make guidelines that you’re going to be following later…I feel like the way you did this, it’s so important because they start connecting like ‘Aha! Yes, now it makes sense’ (L7).

Overall, this style of EBM OSCE was seen as more than just an opportunity to work around barriers. This provided a chance to move forward with a new and potentially less time-intensive method of assessment. Not only is this a great opportunity to address the time barrier, but it could help raise the professional profile of librarians in the eyes of administrators.

### Administrative Factors

#### Professional Respect

Librarians reported that they rarely feel welcomed as assessors of medical students. Additionally, that EBM curriculum and assessments tended to be para-curricular and low priority: ‘I don’t want to say that the college doesn’t care about EBM, but it’s rare - it doesn’t come up very often and things related to exams and placements into residencies - those are the things that kind of drive everything’ (L4). Unfortunately, the overarching feeling was that administrators have yet to understand the value added by HSLs in teaching and assessing EBM competency. Further, librarians are concerned that administrative priorities are more tied to student satisfaction rather than learning outcomes, as reported by one participant: ‘My experience is the thing that’s going to impact the administrators is unfortunately student satisfaction surveys. And EBM tends to be one of the lower rated areas’ (L12). This lack of prioritization is frustrating and feels disrespectful to librarians of the expertise involved in teaching and learning EBM skills:

‘there’s also this lack of understanding about this as a skill-set that requires expertise and repetition and learning over time. We all have advanced degrees and it took us years to get where we are, and I just see over and over: ‘oh, just go talk to a librarian, in 10 minutes you’ll have your skill-set up, or ‘oh, you can have this one hour in the curriculum over four years, and they should know everything’ (L11).

As one librarian responded, it feels possible to make progress in being incorporated more into EBM assessment through improved relationships with medical school leadership: ‘I think, though, you have to have a certain relationship with your school of medicine, in order to be able to talk and convince them to do this’ (L11).

#### Leadership Awareness

Librarians are concerned about the perception that formal EBM education or assessment is no longer needed, as expressed in frustration by one participant: ‘The whole library [has been cut out of the curriculum] because ‘students don’t need it. Students are smart. They know how to find information’ and I’m like ‘no, they really don’t, and they’re putting out pretty crappy research’ (L9). Contributing to being cut out of the curriculum, librarians fear that leaders of the curriculum are often unaware of students’ actual skill level. On the positive side, being made aware of students’ EBM skills performance via direct observation has led to administrators seeking HSL assistance: ‘One of the coordinators saw the student searching on Night on Call and was like ‘oh boy, they’re not doing this well at all’ So her seeing that really opened her eyes and that helped bring us into the curriculum faster than I’ve ever gotten invited’ (L8). Addressing misperceptions through raising the awareness of medical school leaders to actual medical student EBM skills can be eye-opening and transformative. As echoed by one participant: ‘it’s very impactful to see those interactions on video’ (L12).

#### Staffing

Many libraries are short-staffed which leads to severe time limitation for educational activities such as assessment: ‘We only have one librarian for the whole medical school - all 28 departments’ (L1). Libraries that had more librarians on staff dedicated to education (as opposed to research, clinical, or administrative duties) were able to provide more robust assessments and were more deeply integrated into the curriculum. These resource challenges (staffing and time) are critical to address and can hold back libraries from deeper involvement with EBM assessment even when the institution recognizes the need. HSLs often have many other duties beyond providing written formative feedback on assignments. Even for HSLs with educational roles, they often have to split their time among different learner groups: ‘I wish I could do that [EBM assessment] in more of the clerkships and possibly I could, but just personally, I’m also the liaison to the hospital nurses and all the medical residents and fellows, so there might be opportunity that I just don’t have the bandwidth to do’ (L6).

### Curricular Factors

#### Effective Partnership

Librarians felt most effective teaching and assessing EBM when partnering with physician faculty who are engaged and excited about EBM, actively practicing it themselves, and understanding of the librarian’s role in this educational collaboration. This desire is frequently expressed, such as by this participant: ‘If I could partner with faculty and really design something deeper, I think it’ll be much more relevant for them [students] to see that sort of background of why that evidence is necessary’ (L3).

Having physician role models who exemplify the integration of EBM in clinical work is key. While librarians can explain, demonstrate, and assess most of the steps of EBM, they need to work alongside physician role models able to show how they use evidence to inform discussions and decisions in the care of patients. As one participant expressed: ‘They do what the residents do. The residents do a lot of teaching’ (L9). HSLs can in turn support clinician role models to share their process and ‘talk aloud’ how they refine clinical questions, what sources they use, how they search, and what factors lead them to selecting evidence.

Unfortunately, the opposite is also true. When faculty collaborators are not engaged, or non-existent, it erodes the foundation of what is being taught and demonstrated.

‘I feel like when faculty aren’t quite sure how to handle the evidence-based piece they kind of pass it off to the librarian, and then they don’t attend those classes, they’re not there, so they’re not setting the tone for the students. if the faculty member that’s teaching the course isn’t there, then why should the students have to be’ (L1).

#### Time

Shortened and packed curricula continue to impact HSLs ability to keep enough time with medical students to effectively assess EBM: ‘No matter how hard I try, because I’ve tried for over 20 years, we’re getting less and less face to face’ (L9). The trend toward shortening medical schools and adding more content has only made the lack of curricular time worse.

Librarians did see one doorway into getting more curricular time: accreditation requirements. Accreditor visit cycles provide powerful incentive for medical schools to create curricular time for many things, including EBM, but this pressure wanes when deadlines pass: ‘Like all of a sudden they’re really interested in self-directed learning, and we [the librarians] helped a lot with that. And then that [accreditation] happened so they don’t need us anymore to prove that’ (L8).

### Medical Student Factors

#### Learner Self-Awareness

Medical students’ engagement with EBM curricula is highly variable [21,22,23] despite the fact that HSLs work hard to communicate the importance of EBM and its relevance to clinical care. As reported by one participant: ‘I just talked with a student who did this [assignment] a couple of years ago, and that was a big joke for him. Like I looked, and I had given him very poor feedback because he picked some really strange sources, and he said he did it to be funny’ (L8). HSLs are well aware that medical students are highly variable in their ability to practice the basics of EBM, however the students themselves are unaware of their ability or lack thereof. This lack of awareness of their own actual skill level makes collecting and providing formative feedback on actual performance data critical to advocating for a more substantial HSL education role. Providing librarian-mediated opportunities to apply EBM skills, either by conducting literature reviews for research projects or applying skills directly in clinical practice, can make a difference in student awareness and attitude:

‘I’ve had students come back [and say]: ‘I was way ahead of a lot of people in my residency program, because I can search PubMed very well!’ and it’s because they’ve had a research project where they’ve had to do a literature review, and they really have something to hang it on and it’s something they’re interested in. And then if they publish, that’s a whole other level’ (L7).

## Discussion

We aimed to explore the perspective of HSLs on their role in EBM competency assessment of medical students and identifed eight primary themes. The most consistent theme was that librarians are activated. They are ready to assess medical students in more robust and impactful ways. This echoes previous qualitative research on librarian roles [6,8]. There is a growing awareness of the need for consistent and extensible tools. HSLs appreciated a screen-recorded OSCE as a novel way to assess EBM competency because it addresses the perceived lack of consistency in assessment of EBM. This OSCE was seen as an interesting opportunity to set expectations and examine behaviors across schools nationally. Librarians have the skills and interests to engage in EBM assessment, but desire and often lack a pathway to collaborate with others on these types of assessment efforts.

While the librarians were excited about their roles as assessors of EBM and the potential of OSCEs, professional respect, leadership awareness of EBM skills, and limited staff emerged as consistent administrative barriers to implementing assessment plans. Most librarians felt that progress on assessing EBM competency would not be possible until these barriers were addressed by administrators and more resources were secured to support these efforts.

Themes relating to the curriculum are similar to issues that impact other competency content areas. Librarians expressed that having partnership with clinicians who are skilled practitioners of EBM led to the best outcomes driving impactful teaching, assessment, and clinical implementation of EBM skills. Additionally, although curricular time is limited this is certainly not a barrier unique to EBM. At many institutions, EBM content is taught and assessed outside of the standard curriculum. Integrating this work into the formal curricular structure would help alleviate the limited time concerns of librarians and would raise student awareness about the importance of this skill set for their future clinical practice. Despite years of progress, these themes are consistent with earlier findings from Maggio’s work [6,7,11]. Librarians are trying to shoulder the burden of EBM teaching and assessment, but to do so most effectively, strong clinical role models are still needed to reinforce their efforts.

Student self-awareness of the need to gain EBM competency is critical. Most librarians felt that the majority of medical students had limited awareness of their own skill level in practicing EBM. Worse than that, many medical students display poor attitudes when being taught or assessed by librarians. Medical students with poor attitudes were felt to be unaware of the impact of their attitudes both on their own learning and clinical competence and the demoralizing effect on the librarians teaching and assessing them. As has been documented in previous studies, student efficacy and self-awareness are necessary elements to effective EBM teaching and assessment [7,11].

The barriers surfaced in this study should be viewed as opportunities for improvement. Innovations such as self-paced, asynchronous, and recorded modules could be implemented to integrate EBM throughout curricula to help with both time and staffing limitations. EBM assessment programs can be used to raise awareness of the variability in competence for both medical school leadership and students. A well-designed and maintained EBM OSCE can serve as a needs assessment of student competency and a way of monitoring the impact of curricular changes.

EBM OSCEs address many of the barriers discussed, they can facilitate less time-intensive grading, they are familiar and acceptable to students, and librarians and faculty/administrators and they directly connect EBM to clinical work. Once established the assessment information captured in this style of OSCE is likely to provide convincing evidence that will increase buy-in for EBM in the curriculum. Advocacy for library resources is needed. The Night-onCall (NOC) program, from which this OSCE comes, produces comparable data across institutions that need to address the same accreditation EBM requirements. Because of this, it may help individual libraries and groups of librarians obtain institutional buy-in.

## Limitations

There are several limitations of our study. Our sample of librarians drew from diverse locations, types, and sizes of medical schools. However, due to Covid-19, the focus groups were conducted a year after these librarians participated in assessing medical students in an EBM OSCE using the NOC format. This delay could have influenced recollection. Additionally, with most educational programs shifting to asynchronous formats for the year, the librarian’s educational contributions may have been more cut out than usual as schools shifted focus to absolute essentials and decrease staffing levels. This could have influenced HSLs perceived barriers, especially their perceptions of administrative factors. As with all qualitative studies there are many possible ways to interpret the data. The two authors who analyzed the data are practicing medical librarians. They approached this study with reflexivity as to their own roles and the lens they were bringing to the tasks. To help address this limitation, all themes and categories were discussed iteratively with the full group of authors who bring deep expertise in medical education, assessment, and clinical implementation of EBM.

## Conclusion

Librarians identified administrative, curricular, medical student, and librarian factors that acted as barriers and facilitators to effectively assessing EBM behaviors in medical students. Our results point out to medical school leaders the modifiable factors that enable librarians to be more engaged in providing effective feedback. These findings can also help librarians to advocate for themselves and their roles in assessment and highlight the need for novel tools, like a librarian-led EBM OSCE, that can address multiple barriers and create new opportunities for deeper integration of librarians into assessment processes.

## Additional Files

The additional files for this article can be found as follows:

10.5334/pme.1145.s1 Supplementary File 1: Appendix 1. Librarian Focus Group Question Guide.

10.5334/pme.1145.s2 Supplementary File 2: Appendix 2. Coding Data.

## Previous presentations

Presented at EAHIL 2022; Rotterdam, Netherlands; June 2022.
