# Supplementary material for: Librarian-Led Assessment of Medical Students’ Evidence-Based Medicine Competency: Facilitators and Barriers
Source: Perspect Med Educ. 2024 Mar 5;13(1):160–8. doi: 10.5334/pme.1145 (PMC10921970; doi:10.5334/pme.1145)
Supplement: Supplementary File 1: — Appendix 1. Librarian Focus Group Question Guide. [file pme-13-1-1145-s1.pdf]

Questioning Route for Librarian Focus Groups

In this focus group, we're going to be talking about assessing medical student performance of evidence-based medicine behaviors.

Opening Question

How long have you been working as a medical librarian?
What is your current role at your library?

Introductory Question

What are you currently doing in your library to provide feedback for EBM behaviors in medical students?

Transition Question

Are there any gaps or opportunities in your current assessment efforts?

What do you think are important considerations for assessing EBM in medical students?

Further Questions

In our roles, to some degree we are all responsible for assessing EBM behaviors. And we have all had a chance to practice assessment of medical student competence using this EBM video-based OSCE format and rubric.

Based on your own experiences, what advantages or disadvantages do you see in taking this type of approach to EBM assessment? For yourselves as the librarians? For the medical students?

What barriers would you anticipate facing in implementing this activity at our own institution?

Role identity as a facilitator or barrier: Do you see assessment as part of your professional identity? Who should be responsible for doing this kind of assessment? Why or why not? What would help you develop this part of your identity?

Ending Question

Is there anything that we should have talked about, but didn't?
