# Supplementary material for: Librarian-Led Assessment of Medical Students’ Evidence-Based Medicine Competency: Facilitators and Barriers
Source: Perspect Med Educ. 2024 Mar 5;13(1):160–8. doi: 10.5334/pme.1145 (PMC10921970; doi:10.5334/pme.1145)
Supplement: Supplementary File 2: — Appendix 2. Coding Data. [file pme-13-1-1145-s2.pdf]

Appendix Two

The 13 primary codes and 26 sub-codes were applied a total 639 times across the three transcripts.

Table: Code structure developed and used in analysis of focus groups

| Codes | Sub-Codes | Sub-Sub-Codes |
| --- | --- | --- |
| Accreditation | | |
| Barrier | | |
| | Administrative Support | |
| | Limited Staffing | |
| | Time | |
| | Too Many Students | |
| Current State of EBM | | |
| | Aspirations | |
| | Curricular Time | |
| | Multiple Choice Exams | |
| | No Assessment | |
| | OSCE-Style Assessment | |
| | Written Assessment | |
| Curricular Feedback Loop | | |
| EBM Steps | | |
| | Ask (PICO) | |
| | Acquire (Searching) | |
| | Appraise (Critical Appraisal) | |
| | Apply (Clinical Context) | |
| | Assess (Self-Assessment) | |
| Facilitator | | |
| Feedback About EBM OSCEs | | |
| | Pre-Determined Rubric | |
| | Videos of Best Practices (As Feedback) | |
| | Written Feedback | |
| | | Generic Written Feedback |
| | | Individual Written Feedback |
| Physician Champion/Partner | | |
| Role Modeling (For Students) | | |
| Role (Librarian Identity) | | |
| Student EBM Skills | | |
| | Attitude (By Med Students) | |
| | Awareness (By Curriculum Leaders) | |
| | Perceived Student EBM Skills | |

| | |
|---|---|
| | (By Librarians) |
| Technology for Assessment | |
| | Online Score Submission<br>Online Videos (Of Student Behaviors)<br>Software |
